# Reporting of Model Performance and Statistical Methods in Studies That Use Machine Learning to Develop Clinical Prediction Models: Protocol for a Systematic Review

**DOI:** 10.2196/30956

**Published:** 2022-03-03

**Authors:** Colin George Wyllie Weaver, Robert B Basmadjian, Tyler Williamson, Kerry McBrien, Tolu Sajobi, Devon Boyne, Mohamed Yusuf, Paul Everett Ronksley

**Affiliations:** 1 Department of Community Health Sciences Cumming School of Medicine University of Calgary Calgary, AB Canada; 2 Department of Family Medicine Cumming School of Medicine University of Calgary Calgary, AB Canada; 3 Department of Oncology Cumming School of Medicine University of Calgary Calgary, AB Canada; 4 Faculty of Science & Engineering Manchester Metropolitan University Manchester United Kingdom

**Keywords:** machine learning, clinical prediction, research reporting, statistics, research methods, clinical prediction models, artificial intelligence, modeling, eHealth, digital medicine, prediction

## Abstract

**Background:**

With the growing excitement of the potential benefits of using machine learning and artificial intelligence in medicine, the number of published clinical prediction models that use these approaches has increased. However, there is evidence (albeit limited) that suggests that the reporting of machine learning–specific aspects in these studies is poor. Further, there are no reviews assessing the reporting quality or broadly accepted reporting guidelines for these aspects.

**Objective:**

This paper presents the protocol for a systematic review that will assess the reporting quality of machine learning–specific aspects in studies that use machine learning to develop clinical prediction models.

**Methods:**

We will include studies that use a supervised machine learning algorithm to develop a prediction model for use in clinical practice (ie, for diagnosis or prognosis of a condition or identification of candidates for health care interventions). We will search MEDLINE for studies published in 2019, pseudorandomly sort the records, and screen until we obtain 100 studies that meet our inclusion criteria. We will assess reporting quality with a novel checklist developed in parallel with this review, which includes content derived from existing reporting guidelines, textbooks, and consultations with experts. The checklist will cover 4 key areas where the reporting of machine learning studies is unique: modelling steps (order and data used for each step), model performance (eg, reporting the performance of each model compared), statistical methods (eg, describing the tuning approach), and presentation of models (eg, specifying the predictors that contributed to the final model).

**Results:**

We completed data analysis in August 2021 and are writing the manuscript. We expect to submit the results to a peer-reviewed journal in early 2022.

**Conclusions:**

This review will contribute to more standardized and complete reporting in the field by identifying areas where reporting is poor and can be improved.

**Trial Registration:**

PROSPERO International Prospective Register of Systematic Reviews CRD42020206167; https://www.crd.york.ac.uk/PROSPERO/display_record.php?RecordID=206167

**International Registered Report Identifier (IRRID):**

RR1-10.2196/30956

## Introduction

Machine learning is commonly defined as computers learning from data [1], especially when they are not explicitly programmed [2]. There is considerable optimism around the potential benefits of using machine learning approaches in medicine, including for prediction [3-9]. This is in part because of the ever-increasing volume and variety of health care data collected and readily available for clinical and research purposes. Although few prediction models developed using machine learning are currently used in clinical care [10], some researchers believe that machine learning will greatly increase predictive performance relative to more traditional regression techniques and will replace many regression-based prediction models and tasks previously performed by clinicians, such as diagnostic image interpretation [4,10,11]. These increases in performance purport to result in more accurate diagnoses and prognoses for patients and may ultimately improve patient outcomes.

Excitement about these potential benefits has led to a recent increase in the number of publications reporting the use of machine learning approaches for clinical prediction. While several reviews have evaluated the reporting quality of studies that use machine learning for clinical prediction [12-17], they all assessed the studies’ general reporting using items similar to those found on the Transparent Reporting of a Multivariable Prediction Model for Individual Prognosis or Diagnosis (TRIPOD) checklist instead of using machine learning–specific items (eg, related to tuning). We therefore do not know much about the quality of machine learning–specific reporting in studies that use machine learning to develop clinical prediction models. However, there is evidence (albeit limited) suggesting that the reporting of machine learning–specific aspects in these studies is poor [16]. A review by Christodoulou et al [16] evaluated the performance of clinical prediction models developed using machine learning versus regression and found poor reporting of the machine learning tuning methods.

Another review of the reporting of studies that use machine learning for clinical prediction by O’Shea et al [17] assessed the reporting of studies that used convolutional neural networks for the radiological diagnosis of cancer; this review used the CLAIM (Checklist for Artificial Intelligence in Medical Imaging) [18], which was developed for machine learning–based studies. However, most of the CLAIM items are similar to items on non–machine learning checklists and are not unique to machine learning (eg, validation, handling of missing data). Among the unique items, most were specific to artificial neural networks. Two additional reviews evaluated the reporting of studies that used machine learning for clinical prediction [15,16]. However, both these reviews used the TRIPOD checklist to assess reporting quality and therefore the items used were not specific to machine learning. Lastly, a review by Yusuf et al [14] assessed the reporting quality of studies that used machine learning to develop clinical prediction models, but the review focused on the reporting of information about study participants and did not assess reporting of unique machine learning items.

There are no broadly accepted guidelines for the reporting of clinical prediction studies that use machine learning [19-22]. While the TRIPOD statement [23], published in 2015, outlines items that should be reported in all sections of a paper on the development or validation of a clinical prediction model, the specificity of these items as they relate to machine learning methods is limited. Consequently, there is a need for additional guidance on how to report the methods and results of these studies, which differ from traditional regression studies [20,24]. Six reporting guidelines or checklists have been developed for studies that use machine learning approaches [21]: CLAIM [18], MI-CLAIM (Minimum Information About Clinical Artificial Intelligence Modeling) [25], MINIMAR (Minimum Information for Medical Artificial Intelligence Reporting) [26], The Machine Learning Reproducibility Checklist [27], and guidelines by Luo et al [28] and Stevens et al [29]. However, these checklists have substantial limitations, including considerably overlapping with TRIPOD instead of being an extension to TRIPOD. The checklist by Luo et al [28] was the only one to use a Delphi consensus process, a recommended approach [30]. To our knowledge, none of these checklists have become widely used in machine learning clinical prediction research [19-22]. Therefore, the TRIPOD group is developing an extension to the TRIPOD statement for studies that use machine learning—TRIPOD-Artificial Intelligence [31].

The absence of broadly accepted reporting guidelines may contribute to poor reporting. Additionally, studies that use machine learning often have more complex study designs than regression-based clinical prediction model development studies (eg, more complex resampling procedures to accommodate unbiased tuning or model comparison). Complete reporting in clinical prediction research, including research using machine learning, allows for assessment of model validity, assessment of risk of bias in predictive performance, and enables readers to trust and be able to use and externally validate models. Given the lack of reviews assessing machine learning–specific reporting and the evidence of poor reporting of tuning methods, we aim to conduct a systematic review to assess reporting quality using a sample of 100 recently published studies that use machine learning to develop clinical prediction models. The review will consider reporting within 4 key areas where the reporting of machine learning studies is unique: modelling steps, model performance, statistical methods, and presentation of models. The specific objectives of this review are to (1) assess the current reporting quality of machine learning–specific aspects (modelling steps, model performance, statistical methods, and presentation of models) in studies that use machine learning to develop clinical prediction models; (2) evaluate whether reporting quality differs by journal discipline; and (3) identify the most common machine learning algorithms, tuning methods, and internal validation procedures currently used in this field.

## Methods

### Registration and Reporting of Results

This protocol was registered with PROSPERO (registration number CRD42020206167) in September 2020 [32]. The reporting of this protocol follows the Preferred Reporting Items for Systematic Review and Meta-Analysis Protocols statement (Multimedia Appendix 1) [33]. The reporting of the review will follow the PRISMA (Preferred Reporting Items for Systematic Reviews and Meta-Analyses) statement [34]. Ethical considerations do not apply to this review as it will exclusively use data from published studies.

### Search Methods

MEDLINE will be searched without language restrictions via Ovid using a search strategy developed in consultation with a research librarian (Table 1).

**Table 1:** MEDLINE (Ovid) search strategy using Ovid MEDLINE(R) and Ovid MEDLINE Epub Ahead of Print, In-Process and Other Non-Indexed Citations and Daily.

Concept	MEDLINE (Ovid) search terms	Explanation
Supervised machine learning algorithm	exp artificial intelligence/^a^ OR (artificial intelligence OR machine learning OR supervised learning OR statistical learning OR deep learning OR ensemble learning OR regression tree*^b^ OR classification tree* OR “C4.5” OR probability estimation tree* OR random forest* OR support vector machine* OR relevance vector machine* OR artificial neural net* OR deep neural net* OR deep artificial neural net* OR recurrent neural net* OR feedforward neural net* OR feed-forward neural net* OR convolution* neural net* OR perceptron* OR gradient boost* OR adaboost OR k-nearest neighbo* OR nearest neighbo* OR k-NN* OR bayesian network* OR bayesian additive regression tree* OR model tree* OR naive bayes tree* OR bootstrap aggregat* OR hyperparamet* OR hyper-paramet* OR scikit OR tensorflow OR pytorch OR ((neural net* OR decision tree* OR bagging OR boosting OR ensemble* OR tuning OR torch OR CART) AND (cross-valid* OR crossvalid* OR bootstrap* OR predict* OR classifier* OR train OR validat* OR discrimination OR calibration OR ROC curve* OR c-statistic* OR c statistic* or area under the curve OR AUC)) ).mp^c^	Names of common supervised machine learning algorithms (and their implementations) as well as terms related to the use of supervised learning algorithms Terms with non–machine learning meanings as well (eg, bagging) are AND’ed with terms commonly used when discussing prediction models
Animal study	exp animals/^d^ NOT humans/	Animal studies are excluded
Reviews and other non–original research	(review OR meta-analysis OR editorial OR comment OR letter).pt^e^ OR (review OR meta-analysis).ti^f^	Reviews and other publication types not representing original research are excluded
Combining concepts	1 NOT 2 NOT 3	Combining concepts
Limit to 2019	4 AND “2019*”.dp^g^	We are using a random sample of these studies

^a^/: Medical Subject Heading.

^b^*: search term truncation.

^c^.mp: multipurpose (searches titles, abstracts, and author-specified keywords).

^d^exp …/: exploded Medical Subject Heading.

^e^.pt: publication type.

^f^.ti: title.

^g^dp: date of publication.

The machine learning search terms used by Christodoulou et al [16] and the list of machine learning algorithms they identified were used to develop the search terms for this review. An initial search without time limits yielded 141,401 citations. The purpose of this review is to identify common reporting deficiencies in recently published articles and not to conduct an exhaustive assessment of reporting quality of machine learning clinical prediction models over time. We will therefore review citations from only 2019 in a pseudorandom order until we obtain a fixed sample of 100 included articles. This time-restricted sampling approach aims to limit the number of studies in our review because of the impracticality of including all studies that would meet our inclusion criteria (preliminary pilot screening indicates this would be approximately 30,000). Further, this sampling approach is similar to the approach used in other reviews that have aimed to evaluate reporting quality [35]. The number 100 was chosen to obtain a balance between CI width and the time it will take to review articles and extract data. The year 2019 was chosen because it is recent and was not dominated by articles on COVID-19 or influenced by COVID-19–related changes to manuscript writing, reviewing, and publishing practices that may impact reporting quality.

Only one database (MEDLINE) will be used because there are differences in how databases record dates of publication and entry and our review is not meant to be exhaustive. We verified that several key journals that publish machine learning clinical prediction studies are indexed in MEDLINE (eg, *Journal of the American Medical Informatics Association*, *Journal of Medical Internet Research*, *Bioinformatics*, *BMJ Open*, *PLOS One*). Grey literature will not be searched as the objective is to assess reporting quality in peer-reviewed literature.

### Study Selection

Titles and abstracts will be screened independently by 2 reviewers, and abstracts included by either reviewer will proceed to full-text review. Abstracts will be included if they represent peer-reviewed original research where a supervised machine learning algorithm is used to develop a prediction model. The research must have a human health application and the prediction model must be intended for use in clinical practice (specifically, the prediction model must provide a diagnosis, provide a prognosis, or identify candidates for health care interventions). Further details are provided in the complete exclusion criteria list in Textbox 1.

All study designs, medical disciplines, predictor data types (including images, video, and audio), and outcome data types (binary, categorical, ordinal, measured) will be included. Supervised machine learning algorithms create prediction models by learning the relationship between predictors and outcome and are thus most appropriate for this review. In contrast, unsupervised machine learning algorithms identify patterns in data with no specified outcomes; while they can be used as a step in the development of a prediction model, they cannot create prediction models themselves. The reasons for article exclusion will be recorded at both review stages, with criteria applied hierarchically (Textbox 1). After the first 12 abstracts, the reviewers will discuss discrepancies and clarify exclusion criteria.

The same 2 reviewers will independently review full-text articles using the same exclusion criteria, and disagreements will be resolved by discussion and consensus or by consulting a third individual. After the first 7 full-texts, the reviewers will discuss discrepancies and clarify exclusion criteria.

To obtain a random sample of 100 included articles, we will screen citations in the same pseudorandom order at both the title and abstract screening and the full-text review stages. After removing duplicate PubMed identifiers, citations will be sorted by PubMed unique identifier (smallest to largest). In this order, citations will then be assigned a pseudorandomly generated number in R using the rnorm() function with set.seed(3486) and will be sorted by this number (smallest to largest). Title and abstract screening will be conducted using this order until there are 120 studies that will proceed to full-text review. If fewer than 100 of these are included after full-text review, more titles and abstracts will be screened. No full-text articles will be assessed for inclusion after 100 have been included.

A PRISMA flow diagram will be used to describe the stages and reasons for exclusion. Only studies assigned a pseudorandom number less than or equal to the 100th included study will be described in the flow diagram (ie, studies that could not have been included in the review because 100 were obtained will not be described in the flow diagram). The number of records identified by the search strategy without time limits and in 2019 will be reported. Agreement between the reviewers for both the title and abstract screening and the full-text review stages will be assessed by calculating Cohen κ coefficient (including the pilot of 12 abstracts and 7 full-text articles). Google Sheets and Google Forms will be used for study selection, data extraction, and data storage.

**Exclusion criteria (applied in order, with the first-listed applicable criterion recorded as the reason for exclusion).**

**1. The study is not original research.**

- Description
  - Includes reviews, meta-analyses, editorials, comments, or letters

**2. No prediction model is developed.**

- Description
  - Examples are a study that validates a previously developed machine learning prediction model and a robotics study (using machine learning to develop a robot)
  - Studies are also excluded if the primary aim is to identify important predictors or estimate associations between predictors and outcomes rather than to provide individual predictions (develop a prediction model). Studies focused on assessing the incremental value of a new predictor are included
  - Studies are also excluded if their primary objective is to develop a new machine learning methodology or type of machine learning and they place less emphasis on the prediction model(s) developed as a test of the methodology. This exclusion reason is only used in full-text review

- Rationale
  - Most of the reporting items of interest are only applicable to model development studies

**3. No supervised machine learning algorithm is used.**

- Description
  - The study must use at least one supervised machine learning algorithm to develop the prediction model. Studies that only use machine learning to select predictors or process data and not to develop the prediction model are excluded
  - The following are considered machine learning algorithms:
    - Decision tree (classification or regression)
    - Random forest
    - Support vector machine
    - Relevance vector machine
    - Artificial neural network (including single layer perceptron)
    - Boosted tree algorithms (including gradient boosting machines)
    - k-nearest neighbors
    - Bayesian network (only if they learn the graph structure from the data)
    - Bayesian additive regression tree
    - Model tree, including naïve Bayes tree
    - Algorithms employing boosting or bagging
    - Ensemble methods if they include at least one machine learning algorithm listed above
  - The following (by themselves) are not considered machine learning algorithms:
    - Generalized linear model (regression)
    - Regularized (penalized) regression, including lasso, ridge, and elastic net
    - Partial least squares
    - Principal component regression
    - Generalized additive models
    - Regression with splines
    - Multivariate adaptive regression splines
    - Generalized estimating equations
    - Bayesian regression
    - Naïve Bayes
    - Discriminant analysis
    - Genetic algorithms
    - Latent class analysis
    - Fuzzy logic
    - Autoencoder (because not supervised)
  - Studies that only use unsupervised or reinforcement learning are not included because the reporting requirements should be different (no prediction model is developed)
  - Studies that use machine learning to select predictors or process data are excluded because the algorithms are not used to develop the prediction model itself and hence many of the reporting items do not apply (eg, tuning)

- Rationale
  - These algorithms not considered machine learning are mostly regression-based or regression-like and do not learn the structure of the relationship between predictors and outcomes using the data. The implementation, reporting, and risks of bias in these algorithms should be similar to those in regression; the purpose of this review is to assess the reporting quality of machine learning algorithms that may be different or more challenging to report than regression or have different risks of bias

**4. The research does not have human health application.**

- Description
  - Examples are animal study, in vitro study, and research to increase the efficiency of a laboratory procedure

**5. The application is not to provide a diagnosis, provide a prognosis, or identify candidates for health care interventions.**

- Description
  - The prediction model must provide a diagnosis (predict whether a health condition is currently present, screen for a condition, or determine the subtype of a condition), provide a prognosis (predict future health status, outcome, or event), or identify patients who would be better candidates for a health care intervention
  - The prediction model developed must be intended for use in clinical practice either on publishing of the results or on further validation. Similarly, if the aims of the study are mostly etiological and not to develop an accurate prediction model, the study will be excluded
  - Additionally, the prediction model must directly provide a diagnosis or prognosis or identify candidates for health care interventions; models simply aiding humans to do so are excluded (eg, image contouring or segmentation). Models diagnosing image regions individually are included (eg, diagnosing lesions as benign or malignant)
  - The following types of studies will be excluded:
    - Studies that develop case definitions to identify individuals or events in health databases, which would be used for surveillance, quality improvement, or research and not for routine clinical care
    - Studies that develop prediction models to quantify a patient aspect that is not a health condition (eg, height)
    - Studies that develop prediction models to optimize the operation of health care technology or a procedure (eg, a prediction model to determine for which patients setting A should be set to X)

**6. The paper is for a conference.**

- Description
  - Papers published as part of a conference are excluded

**7. The paper is not peer-reviewed.**

**8. The research is reported only in abstract, poster, or presentation form.**

**9. Full text cannot be found.**

**10. Full text is not available in English.**

### Data Extraction

#### Reporting Quality

Reporting quality will be assessed independently by 2 reviewers using a checklist developed in parallel with this review. This checklist includes content derived from existing reporting guidelines, textbooks, and consultations with machine learning experts; a Delphi procedure will not be used. We will then pilot the checklist for 5 studies and revise as needed. Checklist items will be specific to machine learning studies and will not overlap with non–machine learning reporting guidelines (ie, the checklist will be an extension to the TRIPOD checklist). We will not use the TRIPOD adherence assessment form [36] because we are focusing on the machine learning–specific reporting aspects; our review will evaluate these unique reporting items rather than provide a comprehensive reporting assessment of the 100 machine learning studies. This is because we expect the reporting completeness of the aspects not specific to machine learning to be similar to the reporting completeness found in prior reviews of the reporting quality of clinical prediction model studies [24,37,38].

In a draft of the checklist, we identified 4 key areas of study reporting that the checklist will focus on: modelling steps (order and data used for each step), model performance (eg, reporting the performance of each model compared), statistical methods (eg, describing the tuning approach), and presentation of models (eg, specifying the predictors that contributed to the final model). For the assessment of reporting quality, each item can receive 1 of 3 assessments: “reported,” “not reported,” (including incomplete reporting) and “not applicable.” Disagreements will be resolved by discussion and consensus or by consulting a third individual. Agreement on reporting quality assessment between the 2 reviewers will be measured by Cohen κ coefficient for each item and will exclude the pilot of 5 articles.

Reviewers will make their assessments based on what is reported in the text, any supplementary appendices, and the text of any related studies (eg, by the same authors) that are referenced as providing more information on methods used. Reviewers will not contact study authors or look at statistical code to assess reporting quality. Well-reported studies should report these items clearly in words to make it easy for others to understand and critically assess the methods and results. While providing statistical code may be necessary to facilitate understanding and exactly replicate the approach used, in isolation it will not be regarded as sufficient to consider an item “reported.” If there is more than one outcome, analysis, or machine learning algorithm used in the study, reporting quality will be assessed for the primary analysis only. If the primary analysis is not clearly specified, the outcome, analysis, or algorithm mentioned first in the discussion section will be considered primary.

#### Quality Assessment (Risk of Bias)

While the focus of our review will be on reporting quality, we recognize that study quality (ie, risk of bias) may be associated with reporting quality. For this reason, risk of bias within our identified studies will be assessed using the Prediction Model Risk of Bias Assessment Tool (PROBAST) [39]. PROBAST was chosen because it is the only risk of bias tool designed for studies that develop clinical prediction models. Given that we will not have the clinical expertise and context to effectively evaluate prediction models that span multiple medical specialties, we will not assess the first 3 PROBAST domains (participants, predictors, and outcome). Our quality assessment will therefore be limited to the analysis domain within PROBAST. We will not use PROBAST signaling question 4.3 because it requires clinical context to assess or the signaling question 4.9 because it lacks relevance for machine learning studies. We will use a modified version of signaling question 4.8 to improve clarity for machine learning studies (adding the term “data leakage”). We will assign each study a low, high, or unclear risk of bias based on the analysis signaling questions. One reviewer will assess risk of bias using PROBAST’s published form [39], and a second reviewer will verify this information. Disagreements will be resolved by discussion and consensus or by consulting a third individual.

#### Other Data Extraction

In addition to reporting quality and risk of bias, we will extract general study characteristics, prediction model characteristics, tuning method, and internal validation procedures (Textbox 2). These will be extracted by 1 reviewer using a standardized data extraction form and verified by a second reviewer. Disagreements will be resolved by discussion and consensus or by consulting a third individual. We will contact study authors for further information where needed.

**Study characteristics to extract in addition to those for reporting quality and risk of bias.**

**1. General characteristics**

- First author’s last name
- Year of publication
- Title
- Journal
- Journal discipline (clinical, radiology, computer science or engineering, other)
- Country of first author

**2. Prediction model characteristics (if applicable, consider primary outcome or analysis only)**

- Type of outcome predicted (diagnosis of a condition, prognosis of a condition, identification of candidates for a health care intervention)
- Type of data emphasized the most in the title and abstract (coded or structured data, imaging data or video, language [text, audio], genomic or other 'omic data, signal [eg, electrocardiogram])
- Sample size used for model development

**3. Tuning methods (in primary analysis)**

- Were the data used to tune or were fixed or default tuning parameters used? (yes the data were used to tune, no, not mentioned, unclear—if no or not mentioned, the remaining tuning questions are not applicable)
- Search method (grid, random, ad hoc, Bayesian optimization, gradient-based optimization, evolutionary optimization, other, unclear)

**4. Internal validation procedures (in primary analysis)**

- Resampling method, if applicable (data split one or more times, k-fold crossvalidation [including repeated k-fold crossvalidation, leave-one-out crossvalidation], bootstrap, other, unclear)
- Was there a holdout test set not used at any point in the training process? (yes, no, unclear)
- Was nested crossvalidation (or crossvalidation) used in a manner as good as in a holdout test set? (yes, no, unclear)

**5. Other methods**

- Machine learning algorithms used

### Data Synthesis

The primary outcomes are the proportions of included studies that report each of the checklist items across the 4 domains: modelling steps, model performance, statistical methods, and presentation of models. The proportion of applicable items reported per study will also be described using median, first quartile, and third quartile. Items deemed not applicable will be excluded from the proportion denominators. All included studies will be analyzed. As this is a systematic review assessing reporting quality and not synthesizing the findings of individual studies, a meta-analysis will not be performed [35].

We will complete 1 subgroup analysis (objective 2); because of the small sample size, this will be considered exploratory. The proportion of applicable items reported per study will be described by journal discipline (4 categories: clinical, radiology, computer science or engineering, and other). We hypothesize that articles in computer science and engineering journals will report machine learning methods and results more completely, especially the tuning approach.

Ninety-five percent CIs will be reported, including binomial exact CIs for proportions. With 100 included studies, the binomial exact CI widths will be a maximum of 0.20 (at a proportion of 0.5) and a width of 0.18 at a proportion of 0.25, assuming the item is applicable to all studies. One hypothesis test, a Kruskal-Wallis rank sum test, will be performed to determine whether there are differences between the journal disciplines in terms of the proportion of applicable items reported per study. R (version 3.6.2; R Foundation for Statistical Computing) will be used for all analyses [40]. All data collected and code used for this review will be made public via a data repository.

## Results

We completed data analysis in August 2021 and are writing the manuscript. We expect to submit the results to a peer-reviewed journal in early 2022.

## Discussion

Despite the growing use of machine learning for clinical prediction, there is little understanding of the completeness of reporting in these studies, especially reporting aspects unique to machine learning. This review will identify common reporting deficiencies in these areas in 100 recently published studies. Dissemination of these findings to researchers developing machine learning models will increase awareness and the importance of these deficiencies and consequently improve reporting completeness. The novel checklist that is an extension to TRIPOD will also provide an easy way for researchers to improve reporting and allow peer reviewers and editors to assess reporting completeness prior to publication.

We will examine whether reporting quality differs by journal discipline in a subgroup analysis. Results from this secondary objective may highlight key differences in reporting across disciplines and facilitate targeted dissemination or educational activities for disciplines where reporting quality is poor. We will also document the machine learning algorithms currently used in the literature and the accompanying tuning methods and internal validation procedures. Future studies may be able to use this information to identify and improve areas where less preferred approaches (eg, those introducing biases or reducing performance) are often used.

Based on the results of previous reviews on reporting quality [12-14,16,17], we expect to find overall poor reporting quality in our review and possibly a high percentage of studies with high risk of bias. However, we will identify areas where reporting is generally complete and areas where it is lacking in order to improve reporting in this field. Until TRIPOD-Artificial Intelligence [20] is published, the checklist we developed for this review will be the most comprehensive tool available to assess reporting quality of methodological aspects unique to clinical prediction studies that use machine learning. We hope that the checklist we developed and the understanding we gain of areas where reporting is poor will aid the development of TRIPOD-Artificial Intelligence [30].

This review has several strengths. It is the first review to assess the reporting quality of machine learning–specific aspects in published clinical prediction studies that use machine learning. The reporting checklist developed for this review is also novel and focuses on items particularly relevant to models developed using machine learning and not simply on items close to the well-known TRIPOD items [23]. However, this study also has some limitations. First, this review will focus on reporting and will not assess whether the machine learning techniques employed are in line with preferred methodological practices or might introduce bias or reduce performance. The review will only assess risk of bias using the PROBAST analysis domain, which is not specific to machine learning approaches. Second, we chose not to use the more robust Delphi procedure to develop the checklist but chose a smaller and expedited expert consultation. Third, the fixed sample size of 100 studies is relatively small and some estimates of reporting quality, especially in the subgroup analysis, will be imprecise. Fourth, the included studies are likely to be very heterogeneous, especially in terms of the types of machine learning (‘omics to computer vision) and disciplines, which will result in differences in conventions and terminology. This will make it more difficult to assess reporting completeness and may challenge the idea that a single reporting checklist can apply across heterogeneous uses of machine learning for clinical prediction. Findings from this review will help determine if certain types of machine learning may require separate or additional reporting checklists. Fifth, assigning journals to discipline categories requires arbitrary determination, but we believe that our comparison of reporting by discipline remains useful. Finally, it is possible that the search strategy is biased toward better reported studies (ie, studies that use method terms in their abstracts). We have tried to mitigate such bias by keeping our search terms broad.

The use of machine learning in the setting of clinical prediction is growing rapidly, but there is evidence (albeit limited) that suggests that the reporting of machine learning–specific aspects within these studies is poor. This is the first review to assess the reporting of these aspects and enable measurement of current reporting completeness and identification of areas where reporting is lacking. Both the identification of these areas and the novel checklist developed for the review will contribute to more standardized and complete reporting in this field.

## Appendix

Multimedia Appendix 1 PRISMA-P checklist.

## Abbreviations

CLAIM: Checklist for Artificial Intelligence in Medical Imaging
MI-CLAIM: Minimum Information About Clinical Artificial Intelligence Modeling
MINIMAR: Minimum Information for Medical Artificial Intelligence Reporting
PRISMA: Preferred Reporting Items for Systematic Review and Meta-Analysis
PROBAST: Prediction Model Risk of Bias Assessment Tool
TRIPOD: Transparent Reporting of a Multivariable Prediction Model for Individual Prognosis or Diagnosis

## References

[1] Hastie T, Tibsharani R, Friedman J (2009). The Elements of Statistical Learning: Data Mining, Inference, and Prediction. 2nd ed.

[2] Koza J, Bennett IF, Andre D, Keane M (1996). Automated design of both the topologysizing of analog electrical circuits using genetic programming.

[3] Darcy AM, Louie AK, Roberts LW (2016). Machine learning and the profession of medicine. JAMA.

[4] Cabitza F, Rasoini R, Gensini GF (2017). Unintended consequences of machine learning in medicine. JAMA.

[5] Beam AL, Kohane IS (2018). Big data and machine learning in health care. JAMA.

[6] Beam AL, Kohane IS (2016). Translating artificial intelligence into clinical care. JAMA.

[7] Obermeyer Z, Emanuel EJ (2016). Predicting the future - big data, machine learning, and clinical medicine. N Engl J Med.

[8] Verghese A, Shah NH, Harrington RA (2018). What this computer needs is a physician: humanism and artificial intelligence. JAMA.

[9] Chen JH, Asch SM (2017). Machine learning and prediction in medicine - beyond the peak of inflated expectations. N Engl J Med.

[10] Deo RC (2015). Machine learning in medicine. Circulation.

[11] Bates DW, Saria S, Ohno-Machado L, Shah A, Escobar G (2014). Big data in health care: using analytics to identify and manage high-risk and high-cost patients. Health Aff (Millwood).

[12] Andaur Navarro CL, Damen JAA, Takada T, Nijman SWJ, Dhiman P, Ma J, Collins GS, Bajpai R, Riley RD, Moons KGM, Hooft L (2021). Completeness of reporting of clinical prediction models developed using supervised machine learning: a systematic review. medRxiv.

[13] Dhiman P, Ma J, Navarro Constanza Andaur, Speich B, Bullock G, Damen JA, Kirtley S, Hooft L, Riley RD, Van Calster B, Moons KG, Collins GS (2021). Reporting of prognostic clinical prediction models based on machine learning methods in oncology needs to be improved. J Clin Epidemiol.

[14] Yusuf M, Atal I, Li J, Smith P, Ravaud P, Fergie M, Callaghan M, Selfe J (2020). Reporting quality of studies using machine learning models for medical diagnosis: a systematic review. BMJ Open.

[15] Nagendran M, Chen Y, Lovejoy CA, Gordon AC, Komorowski M, Harvey H, Topol EJ, Ioannidis JPA, Collins GS, Maruthappu M (2020). Artificial intelligence versus clinicians: systematic review of design, reporting standards, and claims of deep learning studies. BMJ.

[16] Christodoulou E, Ma J, Collins GS, Steyerberg EW, Verbakel JY, Van Calster B (2019). A systematic review shows no performance benefit of machine learning over logistic regression for clinical prediction models. J Clin Epidemiol.

[17] O'Shea Robert J, Sharkey AR, Cook GJR, Goh V (2021). Systematic review of research design and reporting of imaging studies applying convolutional neural networks for radiological cancer diagnosis. Eur Radiol.

[18] Mongan J, Moy L, Kahn CE (2020). Checklist for Artificial Intelligence in Medical Imaging (CLAIM): a guide for authors and reviewers. Radiol Artif Intell.

[19] El Naqa I, Ruan D, Valdes G, Dekker A, McNutt T, Ge Y, Wu QJ, Oh JH, Thor M, Smith W, Rao A, Fuller C, Xiao Y, Manion F, Schipper M, Mayo C, Moran JM, Ten Haken R (2018). Machine learning and modeling: Data, validation, communication challenges. Med Phys.

[20] Collins GS, Moons KGM (2019). Reporting of artificial intelligence prediction models. Lancet.

[21] Ibrahim H, Liu X, Denniston AK (2021). Reporting guidelines for artificial intelligence in healthcare research. Clin Exp Ophthalmol.

[22] Liu X, Faes L, Kale AU, Wagner SK, Fu DJ, Bruynseels A, Mahendiran T, Moraes G, Shamdas M, Kern C, Ledsam JR, Schmid MK, Balaskas K, Topol EJ, Bachmann LM, Keane PA, Denniston AK (2019). A comparison of deep learning performance against health-care professionals in detecting diseases from medical imaging: a systematic review and meta-analysis. Lancet Digit Health.

[23] Collins GS, Reitsma JB, Altman DG, Moons KGM (2015). Transparent reporting of a multivariable prediction model for individual prognosis or diagnosis (TRIPOD): the TRIPOD statement. BMJ.

[24] Heus P, Damen JAAG, Pajouheshnia R, Scholten RJPM, Reitsma JB, Collins GS, Altman DG, Moons KGM, Hooft L (2018). Poor reporting of multivariable prediction model studies: towards a targeted implementation strategy of the TRIPOD statement. BMC Med.

[25] Norgeot B, Quer G, Beaulieu-Jones BK, Torkamani A, Dias R, Gianfrancesco M, Arnaout R, Kohane IS, Saria S, Topol E, Obermeyer Z, Yu B, Butte AJ (2020). Minimum information about clinical artificial intelligence modeling: the MI-CLAIM checklist. Nat Med.

[26] Hernandez-Boussard T, Bozkurt S, Ioannidis J, Shah N (2020). MINIMAR (MINimum Information for Medical AI Reporting): Developing reporting standards for artificial intelligence in health care. J Am Med Inform Assoc.

[27] Pineau J, Vincent-Lamarre P, Sinha K (2021). Improving reproducibility in machine learning research (a report from the NeurIPS 2019 Reproducibility Program). J Mach Learn Res.

[28] Luo W, Phung D, Tran T, Gupta S, Rana S, Karmakar C, Shilton A, Yearwood J, Dimitrova N, Ho TB, Venkatesh S, Berk M (2016). Guidelines for developing and reporting machine learning predictive models in biomedical research: a multidisciplinary view. J Med Internet Res.

[29] Stevens LM, Mortazavi BJ, Deo RC, Curtis L, Kao DP (2020). Recommendations for reporting machine learning analyses in clinical research. Circ Cardiovasc Qual Outcomes.

[30] Moher D, Schulz KF, Simera I, Altman DG (2010). Guidance for developers of health research reporting guidelines. PLoS Med.

[31] Collins GS, Dhiman P, Andaur Navarro CL, Ma J, Hooft L, Reitsma JB, Logullo P, Beam AL, Peng L, Van Calster B, van Smeden M, Riley RD, Moons KG (2021). Protocol for development of a reporting guideline (TRIPOD-AI) and risk of bias tool (PROBAST-AI) for diagnostic and prognostic prediction model studies based on artificial intelligence. BMJ Open.

[32] University of York Centre for Reviews and Dissemination (2022). PROSPERO. PROSPERO: international prospective register of systematic reviews.

[33] Moher D, Shamseer L, Clarke M, Ghersi D, Liberati A, Petticrew M, Shekelle P, Stewart LA, PRISMA-P Group (2015). Preferred reporting items for systematic review and meta-analysis protocols (PRISMA-P) 2015 statement. Syst Rev.

[34] Moher D, Liberati A, Tetzlaff J, Altman DG, PRISMA Group (2009). Preferred reporting items for systematic reviews and meta-analyses: the PRISMA statement. BMJ.

[35] Moher D, Tetzlaff J, Tricco AC, Sampson M, Altman DG (2007). Epidemiology and reporting characteristics of systematic reviews. PLoS Med.

[36] Heus P, Damen JAAG, Pajouheshnia R, Scholten RJPM, Reitsma JB, Collins GS, Altman DG, Moons KGM, Hooft L (2019). Uniformity in measuring adherence to reporting guidelines: the example of TRIPOD for assessing completeness of reporting of prediction model studies. BMJ Open.

[37] Takemura T, Kataoka Y, Uneno Y, Otoshi T, Matsumoto H, Tsutsumi Y, Tsujimoto Y, Yuasa M, Yoshioka T, Wada H (2018). The reporting quality of prediction models in oncology journals: a systematic review. Annals of Oncology.

[38] Wynants L, Kent DM, Timmerman D, Lundquist CM, Van Calster B (2019). Untapped potential of multicenter studies: a review of cardiovascular risk prediction models revealed inappropriate analyses and wide variation in reporting. Diagn Progn Res.

[39] Wolff RF, Moons KG, Riley RD, Whiting PF, Westwood M, Collins GS, Reitsma JB, Kleijnen J, Mallett S, PROBAST Group† (2019). PROBAST: a tool to assess the risk of bias and applicability of prediction model studies. Ann Intern Med.

[40] R Core Team R: The R Project for Statistical Computing. R: A Language and Environment for Statistical Computing.

